# EPA or DHA enhanced oxidative stress and aging protein expression in brain of d-galactose treated mice

**DOI:** 10.7603/s40681-016-0017-1

**Published:** 2016-08-12

**Authors:** Yuan-Man Hsu, Mei-Chin Yin

**Affiliations:** 1 Department of Biological Science and Technology, China Medical University, 404 Taichung, Taiwan; 2 Department of Nutrition, China Medical University, 91, Hsueh-shih Rd., 404 Taichung, Taiwan

**Keywords:** Brain aging, DHA, EPA, NADPH oxidase, d-Galactose

## Abstract

**Background:**

Effects of eicosapentaenoic acid (EPA, 20:5) and docosahexaenoic acid (DHA, 22:6) upon fatty acid composition, oxidative and inflammatory factors and aging proteins in brain of d-galactose (DG) treated aging mice were examined.

**Methods:**

Each fatty acid at 7 mg/kg BW/week was supplied for 8 weeks. Brain aging was induced by DG treatment (100 mg/kg body weight) *via* daily subcutaneous injection for 8 weeks.

**Results:**

DG, EPA and DHA treatments changed brain fatty acid composition. DG down-regulated brain Bcl-2 expression and up-regulated Bax expression. Compared with DG groups, EPA and DHA further enhanced Bax expression. DG decreased glutathione content, increased reactive oxygen species (ROS) and oxidized glutathione (GSSG) production, the intake of EPA or DHA caused greater ROS and GSSG formation. DG treatments up-regulated the protein expression of p47^phox^ and gp91^phox^, and the intake of EPA or DHA led to greater p47^phox^ and gp91^phox^ expression. DG increased brain prostaglandin E2 (PGE2) levels, and cyclooxygenase (COX)-2 expression and activity, the intake of EPA or DHA reduced brain COX-2 activity and PGE_2_ formation. DG enhanced brain p53, p16 and p21 expression. EPA and DHA intake led to greater p21 expression, and EPA only caused greater p53 and p16 expression. Conclusion: These findings suggest that these two PUFAs have toxic effects toward aging brain.

## 1. Introduction

Eicosapentaenoic acid (EPA, 20:5) and docosahexaenoic acid (DHA, 22:6) are n-3 polyunsaturated fatty acids (PUFAs), and their major dietary sources are sea foods. It has been reported that EPA and DHA could improve hepatic lipid metabolism and prevent cardiovascular events [1, 2]. The studies of Sublette *et al*. [3] and Song *et al*. [4] revealed that these PUFAs could alleviate the progression of neurodegenerative disorders such as depression and Alzheimer’s disease. The American Heart Association suggests healthy person to consume at least two servings fish per week, which results in EPA and DHA weekly intake at 400- 500 mg [5]. Although those previous studies support the healthy benefits of EPA and DHA, the safety and even toxic effects of these two PUFAs are also paid attention. Chen *et al*. [6] indicated that fish omega-3 fatty acids supplementation decreased liver glutathione (GSH) level and increased liver fibrosis in bile duct-ligated rats. The study of Yang *et al*. [7] revealed that DHA exhibited detrimental effects such as increasing malondialdehyde production, lowering GSH content in post-ischemic progression and brain injury. So far, the impact of EPA and DHA upon brain under aging condition remains unknown.

Mitochondrial apoptotic pathway, mainly mediated by BCL family proteins, is involved in neurons death and responsible for pathological development of brain aging. This pathway includes pro-apoptotic molecules such as Bax, and anti-apoptotic molecules such as Bcl-2 [8]. NADPH oxidase complex is a key regulator for the generation of free radicals, especially reactive oxygen species (ROS), which contributes to decrease neurogenesis and impair brain functions [9, 10]. In addition, the activation of aging associated signaling pathways including nuclear factor erythroid-derived 2-like 2 (Nrf2), nuclear factor kappaB (NF- κB), heme oxygenase-1 (HO-1) and cyclooxygenase (COX)-2 initiates mitochondrial dysfunction and stimulates the release of oxidants and inflammatory cytokines such as interleukin (IL)-6, tumor necrosis factor (TNF)-α and prostaglandin E2 (PGE_2_) [11-13]. So far, the impact of EPA or DHA upon these factors or signaling pathways in aging brain, often occurred in elderly people, remains unclear. On the other hand, Sirtuin 1, p53, p16 and p21 are aging-associated proteins. Sirtuin 1, a nicotinamide adenine dinucleotide-dependent deacetylase, participates normal cognitive functions and prevents aging-associated neuronal degeneration [14]. p53, a transcription factor, controls cell-cycle and cellular stress responses, and its activation mediates cellular senescence [15, 16]. The expression of p16 and p21 increases with aging in human tissues including brain, which facilitates senescence through negatively regulating the cell cycle [17, 18]. Thus, any agents with the capability to modulate these aging-associated proteins in brain may delay or promote senescence.

D-galactose (DG)-induced neuro-pathological alteration has been applied as an aging model because DG over-supply induces apoptotic, oxidative and inflammatory stress in the nervous system [19, 20]. In our present study, DG treated aging mice were used to investigate the influence of DHA or EPA upon brain. The effects of these two PUFAs upon fatty acid composition, oxidative and inflammatory factors and aging proteins in brain were examined. The influence of these PUFAs upon protein expression of Bcl-2, Bax, NAPDH oxidase, Nrf2, COX-2, p53, p21 and NF-κB in brain were also determined in order to elucidate the possible action modes of EPA and DHA.

## 2. Materials and methods

### 2.1. Materials

Three n-3 fatty acids, α-linolenic acid (ALA, 18:3, 99.5%), DHA (99%) and EPA (99%), were purchased from Sigma Chemical Co. (St. Louis, MO, USA). ALA, an essential fatty acid, is a shorter chain fatty acid than EPA or DHA. It was used in this study for comparison. DG (99.5%) was bought from Wako Pure Chemical Co. (Tokyo, Japan).

### 2.2. Animals and diet

Three-week old male Balb/cA mice were obtained from National Laboratory Animal Center (Taipei City, Taiwan). Mice were housed on a condition with 12-h light/dark. Mouse standard diet and water were consumed *ad libitum*. The use of mice was reviewed and approved by China Medical University animal care and use committee, and permission number was 104-305.

### 2.3. Experimental design

Mice at 7-month old were used for experiments. Mice were divided into non-DG and DG groups. Non-DG group was used as normal group. DG groups were treated with DG (100 mg/kg body weight) *via* daily subcutaneous injection for 8 weeks. Song *et al*. [21] reported that 8-wk DG treatment induced 24 months aging. Therefore, 7-month old mice with 8-wk DG injection led to mice at 31-month old, which was similar to 80-year old human [22]. These DG mice were further divided into four sub-groups. One DG group without fatty acid intake was used for control. The other three DG groups were treated by ALA, EPA or DHA at 3.5 mg/kg BW/time and twice per week. The intake of each fatty acid was 7 mg/kg BW/week in mice, which was equal to 490 mg/ week for a 70-kg adult. After 8-wk DG and fatty acid treatments, mice were sacrificed by decapitation. Brain was collected, and 100 mg brain was homogenized in 2 ml of ice cold phosphate buffer saline (PBS, pH 7.2). Protein concentration was quantified by an assay kit purchased from Pierce Biotechnology Inc. (Rockford, IL, USA), in which bovine serum albumin acted as a standard. Plasma activity of alanine aminotransferase (ALT) and aspartate aminotransferase (AST) was examined for liver functions, which were measured by assay kits (Randox Laboratories Ltd., Crumlin, UK).

### 2.4. Brain triglyceride (TG) and cholesterol measurements

One ml brain homogenate was mixed with chloroform/methanol solution (2:1, v/v). After vigorously shaking, chloroform layer was collected and followed by concentrating with a rotary evaporator. After reacting with isopropanol containing 10% Triton X-100, Wako E-Test kits (Wako Pure Chemical, Osaka, Japan) were used to determine TG and TC content. HDL level was determined by an ELISA kit (Pointe Scientific, Inc., Canton, MI), and LDL level was calculated according to the following equation: total cholesterol – HDL cholesterol – (TG/5).

### 2.5. Brain fatty acid composition

A HP5890 gas chromatography equipped with FID and a 30-m Omegawax capillary column (Supelco Chromatography Products, Bellefonte, PA, USA) was used for analyzing fatty acid composition. Each fatty acid was measuring by detecting the areas under identified peaks. Data are shown as percentage of total fatty acids.

### 2.6. Determination of oxidative factors

Brain ROS level was measured by using an oxidation sensitive dye, 2’, 7’-dichlorofluorescein diacetate (DCFH-DA). Briefly, 100 ml brain homogenate was reacted with 100 ml DCFH-DA (2 mg/ml) for 30 min at 37°C. Fluorescence was measured at 488 nm and 525 nm for excitation and emission, respectively, by a fluorescence plate reader. Data are expressed as relative fluorescence unit (RFU)/mg protein. Brain GSH and GSSG levels (nmol/mg protein) were analyzed by commercial colorimetric kits (OxisResearch, Portland, OR, USA). Assay kits purchased from EMD Biosciences (San Diego, CA, USA) were used to determine the activity (U/mg protein) of glutathione peroxidase (GPX) and glutathione reductase (GR).

### 2.7. Measurement of inflammatory factors

Partial brain tissue was mixed with 10 mM Tris-HCl solution (pH 7.4) containing 1 mM EDTA, 2 M NaCl, 0.01% Tween 80, 1 mM phenylmethylsulfonyl fluoride, and followed by homogenized and centrifuged at 9000 xg for 40 min at 4°C. Then, supernatant was used for determining IL-1β, IL-6 and TNF-α levels by using cytoscreen immunoassay kits (BioSource International, Camarillo, CA, USA). Brain PGE_2_ level and COX-2 activity were assayed using kits obtained from Cayman Chemical Co. (Ann Arbor, MI, USA). COX-2 activity was quantified by monitoring the absorbance at 590 nm, which meant the formation of oxidized N, N, N’, N’-tetramethyl-p-phenylenediamine.

### 2.8. NF-κB binding activity assay

The nuclear extract of brain tissue was used for NF-κB p50/65 DNA binding activity assay, which was measured by a kit (Chemicon International Co., Temecula, CA, USA). The binding of activated NF-κB was processed by adding 3, 3′, 5, 5′- tetramethylbenzidine as a substrate, a primary antibody against NF-κB p50/p65 and a secondary horseradish peroxidase-conjugated antibody. The change in absorbance at 450 nm was recorded. Data are shown in relative optical density (OD)/mg protein.

**Table 1:** 

	normal	DG	DG + ALA	DG + EPA	DG + DHA
BW	32.5 ± 2.1	31.8 ± 1.7	32.7 ± 1.4	30.9 ± 2.3	31.5 ± 1.8
WI	2.2 ± 0.7	2.0 ± 0.8	2.3 ± 0.4	2.4 ± 0.5	2.2 ± 0.6
FI	2.3 ± 0.4	2.5 ± 0.6	2.2 ± 0.7	2.4 ± 0.3	2.0 ± 0.2
Brain	0.51 ± 0.04	0.48 ± 0.07	0.53 ± 0.05	0.46 ± 0.06	0.49 ± 0.08
ALT	28 ± 4	26 ± 2	30 ± 5	27 ± 3	32 ± 5
AST	31 ± 4	32 ± 3	28 ± 2	29 ± 4	30 ± 3

**Table 2:** 

	normal	DG	DG + ALA	DG + EPA	DG + DHA
TG	268 ± 18^b^	274 ± 9^b^	263 ± 12^b^	217 ± 10^a^	210 ± 7^a^
TC	214 ± 13^b^	205 ± 10^b^	196 ± 14^b^	150 ± 8^a^	146 ± 11^a^
HDL	68 ± 5^a^	71 ± 7^a^	66 ± 4^a^	69 ± 6^a^	72 ± 3^a^
LDL	113 ± 10^b^	108 ± 6^b^	104 ± 8^b^	72 ± 4^a^	67 ± 7^a^

^a, b^Means in a row without a common letter differ, *P* < 0.05.

### 2.9. Western blot analysis

Brain tissue was homogenized in protease-inhibitor cocktail (1:1000) containing buffer purchased from Sigma-Aldrich Chemical Co. (St. Louis, MO, USA) and 0.5% Triton X-100. This homogenate was then mixed with solution containing 60 mM Tris-HCl, 2% SDS, and 2% β-mercaptoethanol (pH 7.2), and further boiled for 5 min. Forty μg protein sample was applied to SDS-PAGE, and followed by transferring to a nitrocellulose membrane for 60 min. After treating with a 5% nonfat milk solution for another 60 min, membranes were further incubated with mouse monoclonal antibody against Bcl-2, Bax (1:2000), p47^phox^, gp91^phox^, Nrf2, HO-1, COX-2, NF-κB (1:1000), Sirtuin 1, p53, p21 and p16 (1:500) at 4ºC for 24 hr, and followed by reacting with a horseradish peroxidase conjugated antibody for 3.5 hr at room temperature. These monoclonal antibodies were purchased from Boehringer-Mannheim Co. (Indianapolis, IN, USA). The image of formed blot was processed by autoradiography, and quantified by normalized to GAPDH.

### 2.10. Statistical analysis

Data of each measurement were obtained from 10 mice (n = 10), and data were expressed as mean ± standard deviation (SD). Statistical analysis was processed using one-way analysis of variance. *Post-hoc* comparisons were also carried out by Dunnett’s *t*-test. *P* value < 0.05 was defined as statistically significant.

## 3. Results

### 3.1. EPA or DHA lowered brain TG and TC content

As shown in Table 1, DG treatment and 3 fatty acids intake did not affect body weight, water intake, feed intake, brain weight, plasma ALT and AST activities (*P* > 0.05). Compared with normal groups, DG treatment did not change brain TG, TC, HDL and LDL content (Table 2, *P* > 0.05); however, the intake of EPA or DHA, not ALA, decreased brain TG, TC and LDL content (*P* < 0.05).

### 3.2. EPA or DHA changed brain fatty acid composition

Compared with normal groups, DG treatment increased saturated fatty acid (SFA) content and decreased monounsaturated fatty acid (MFA) content in brain (Table 3, P < 0.05). The intake of ALA, EPA or DHA increased their content in brain, respectively; and lowered SFA and increased PUFA content in brain (*P* < 0.05). DG treatment decreased 18:3 and increased 20:4 levels; the intake of ALA, EPA or DHA reversed these changes (*P* < 0.05). DG caused 20:1 and 22:6 undetectable; but EPA or DHA intake increased the levels of these two fatty acids in brain (*P* < 0.05).

### 3.3. EPA or DHA enhanced brain apoptotic and oxidative stress

**Figure 1:**
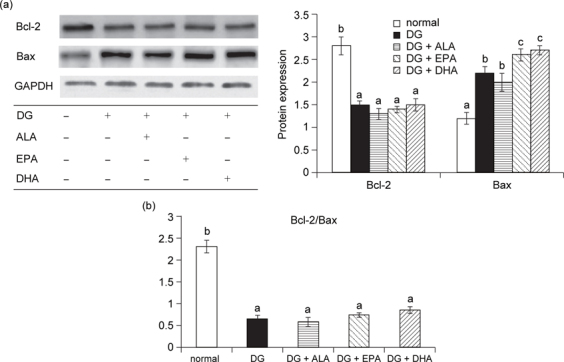

**Figure 2:**
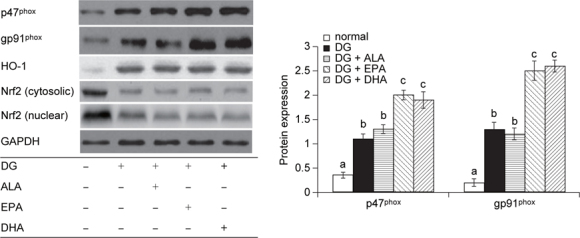

DG down-regulated Bcl-2 expression and up-regulated Bax expression (Figure 1a, *P* < 0.05). Compared with DG groups, EPA and DHA significantly enhanced Bax expression (*P* < 0.05). DG reduced the ratio of Bcl-2/Bax (Figure 1b, *P* < 0.05); and ALA, EPA or DHA intake did not further affect this ratio (*P* > 0.05). DG increased ROS and GSSG production, decreased GSH content, and lowered GPX, GR and catalase activities in brain (Table 4, *P* < 0.05). Compared with DG groups, the intake of EPA or DHA, not ALA, caused greater ROS and GSSG formation and less GR activity (*P* < 0.05). As shown in Figure 2, DG treatments up-regulated the protein expression of p47^phox^, gp91^phox^, and HO-1 expression, and down-regulated cytosolic and nuclear Nrf2 expression (*P* < 0.05). Compared with DG groups, the intake of EPA or DHA led to greater p47^phox^ and gp91^phox^ expression (*P* < 0.05). But, three test fatty acids did not further affect HO-1 and Nrf2 expression (*P* > 0.05).

**Table 3:** 

	normal	DG	DG + ALA	DG + EPA	DG + DHA
14:0	0.83 ± 0.06^a^	0.77 ± 0.08^a^	1.02 ± 0.12^a^	0.58 ± 0.06^a^	0.67 ± 0.09^a^
16:0	11.21 ± 1.11^a^	12.86 ± 1.05^b^	10.33 ± 0.96^a^	11.09 ± 0.82^a^	10.82 ± 1.24^a^
18:0	26.84 ± 2.24^a^	28.55 ± 1.94^b^	27.43 ± 2.08^a^	25.8 ± 1.34^a^	26.09 ± 2.33^a^
Total SFA	38.88 ± 3.81^a^	42.18 ± 2.03^b^	38.78 ± 3.07^a^	37.48 ± 2.62^a^	37.58 ± 1.59^a^
16:1	3.92 ± 0.32^a^	3.25 ± 0.08^a^	3.84 ± 0.47^a^	3.44 ± 0.29^a^	3.12 ± 0.16^a^
18:1	28.86 ± 2.34^a^	28.07 ± 1.67^a^	27.15 ± 2.48^a^	28.56 ± 1.38^a^	27.45 ± 1.89^a^
20:1	1.08 ± 0.07^b^	-^a^	0.86 ± 0.05^b^	0.92 ± 0.1^b^	1.13 ± 0.08^b^
Total MFA	33.86 ± 3.66^b^	31.32 ± 2.9^a^	31.85 ± 2.07^a^	32.92 ± 2.58^b^	31.70 ± 2.71^a^
18:2	20.07 ± 1.07^a^	19.41 ± 0.99^a^	22.26 ± 1.42^b^	20.11 ± 2.05^a^	20.66 ± 1.74^a^
18:3, ALA	2.13 ± 0.38^b^	1.12 ± 0.21^a^	3.02 ± 0.29^c^	2.18 ± 0.17^b^	2.47 ± 0.33^b^
20:4	2.28 ± 0.27^b^	3.73 ± 0.38^c^	1.37 ± 0.18^a^	2.03 ± 0.25^b^	2.10 ± 0.3^b^
20:5, EPA	0.68 ± 0.09^a^	0.42 ± 0.07^a^	0.82 ± 0.04^a^	2.39 ± 0.31^b^	1.55 ± 0.19^b^
22:6, DHA	0.56 ± 0.07^b^	-^a^	-^a^	1.17 ± 0.11^c^	2.14 ± 0.29^d^
Total PUFA	25.72 ± 2.24^a^	24.68 ± 1.95^a^	27.47 ± 2.07^b^	27.88 ± 2.35^b^	28.92 ± 2.41^b^

-Means too low to be detected.

^a-d^Means in a row without a common letter differ, *P* < 0.05.

**Table 4:** 

	normal	DG	DG + ALA	DG + EPA	DG + DHA
ROS	0.18 ± 0.06^a^	1.91 ± 0.17^b^	1.87 ± 0.13^b^	2.29 ± 0.11^c^	2.36 ± 0.18^c^
GSSG	0.11 ± 0.08^a^	1.42 ± 0.13^b^	1.38 ± 0.2^b^	1.75 ± 0.15^c^	1.86 ± 0.07^c^
GSH	94 ± 5^b^	43 ± 2^a^	46 ± 4^a^	40 ± 3^a^	35 ± 4^a^
GPX	23.1 ± 2.0^b^	10.8 ± 1.1^a^	11.3 ± 1.4^a^	10.6 ± 0.9^a^	11.6 ± 1.6^a^
GR	21.8 ± 1.7^c^	12.1 ± 0.8^b^	11.9 ± 1.2^b^	9.8 ± 0.6^a^	9.5 ± 0.9^a^
catalase	24.0 ± 1.9^b^	13.5 ± 1.3^a^	13.8 ± 1.0^a^	12.7 ± 1.4^a^	12.1 ± 0.8^a^

^a-c^Means in a row without a common letter differ, *P* < 0.05.

**Table 5:** 

	normal	DG	DG + ALA	DG + EPA	DG + DHA
IL-1β	0.97 ± 0.08^a^	3.31 ± 0.14^b^	3.34 ± 0.06^b^	3.27 ± 0.11^b^	3.21 ± 0.1^b^
IL-6	1.03 ± 0.05^a^	3.23 ± 0.12^b^	3.08 ± 0.15^b^	3.13 ± 0.11^b^	3.04 ± 0.09^b^
TNF-α	0.91 ± 0.06^a^	3.87 ± 0.14^b^	3.83 ± 0.2^b^	3.91 ± 0.17^b^	3.94 ± 0.15^b^
PGE_2_	973 ± 78^a^	2184 ± 149^d^	1508 ± 118^b^	1788 ± 95^c^	1762 ± 104^c^
COX-2	0.24 ± 0.06^a^	1.90 ± 0.18^d^	1.15 ± 0.11^b^	1.45 ± 0.13^c^	1.52 ± 0.09^c^

^a-c^Means in a row without a common letter differ, *P* < 0.05.

### 3.4. EPA or DHA lowered brain COX-2 activity and PGE_2_ formation

**Figure 3:**
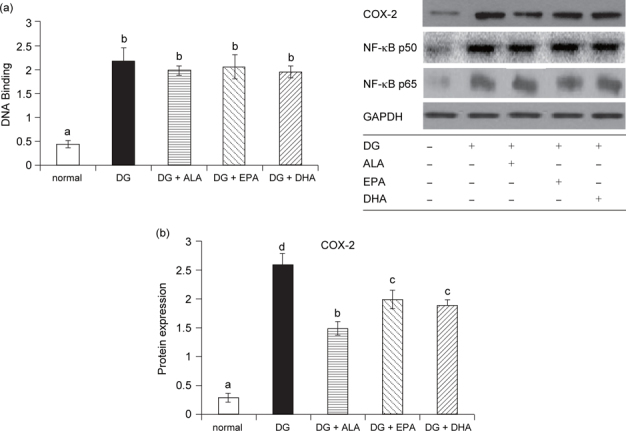

DG increased brain IL-1β, IL-6, TNF-α and PGE2 levels, and COX-2 activity (Table 5, *P* < 0.05). Compared with DG groups, the intake of ALA, EPA or DHA led to similar levels of IL-1β, Il-6, TNF-α (*P* > 0.05). However, ALA, EPA or DHA treatments reduced brain COX-2 activity and PGE2 formation (*P* < 0.05). DG increased NF-κB p50/65 DNA binding activity (Figure 3a, *P* < 0.05) and up-regulated protein expression of NF-κB p50, NF-κB p65 and COX-2 (Figure 3b, *P* < 0.05). Compared with DG groups, the intake of ALA, EPA or DHA did not further alter NF- κB p50/65 DNA binding activity and the expression of NF-κB p50 and NF-κB p65 (*P* > 0.05); however, ALA, EPA or DHA treatments suppressed brain COX-2 expression (*P* < 0.05).

**Figure 4:**
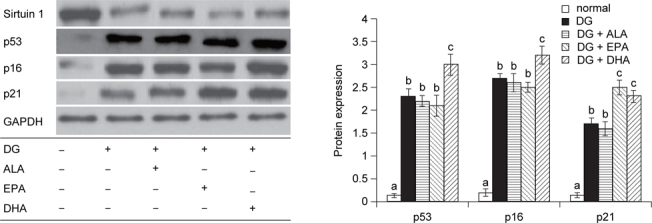

### 3.5. EPA or DHA altered brain aging protein expression

As shown in Figure 4, DG suppressed Sirtuin 1 expression and enhanced p53, p16 and p21 expression (*P* < 0.05). Compared with DG groups, 3 test fatty acids did not change Sirtuin 1 expression (*P* > 0.05). But, EPA and DHA intake led to greater p21 expression, and EPA only caused greater p53 and p16 expression (*P* < 0.05).

## 4. Discussion

Our present study is the first one to examine the influence of DG upon brain fatty acid composition, and we found that DG decreased brain MFAs, but increased SFAs. These changes may impair brain functions because SFAs display negative actions upon memory, promote cognitive decline, and cause apoptosis [23, 24].

Our major purpose was to investigate the influence of dietary ALA, EPA or DHA upon brain in DG induced aging model, in which ALA was used for comparison. The intake of each fatty acid at 7 mg/kg BW/week in mice was equal to 490 mg/week for a 70-kg adult. This used dose was based on the recommendation from The American Heart Association, two fish servings per week offers 400-500 mg EPA and DHA [5]. Our data revealed that ALA intake lowered brain SFAs and arachidonic acid (AA, 20:4) levels, but did not affect other measurements when compared with DG groups. Thus, ALA at this dose seems safe and able to alleviate brain inflammation through declining AA cascade. However, we found that EPA or DHA at the same dose as ALA markedly decreased brain content of LDL, SFAs and AA. The accumulation of LDL and SFAs are highly related to the development of stroke, atherosclerosis, thrombosis or even neurodegenerative diseases [25, 26]. Since EPA or DHA substantially lowered brain LDL and SFAs levels under aging condition, the intake of these two PUFAs may reduce the occurrence of the above disorders. On the other hand, EPA or DHA intake increased brain PUFAs content, ROS and GSSG production, and protein expression of Bax, p47^phox^, gp91^phox^, p53, p16 and p21. These findings indicated that EPA or DHA at this dose displayed apoptotic and oxidative toxicity toward brain.

Bax is a pro-apoptotic molecule, and we found EPA or DHA intake increased brain Bax expression although they did not raise Bcl-2/Bax ratio. These data suggested that higher doses and/or longer time of EPA and/or DHA intake might enhance brain apoptotic stress. The activation of NADPH oxidase is an important source of ROS in neurons, and responsible for brain oxidative injury [27]. EPA or DHA intake effectively promoted brain protein expression of p47^phox^ and gp91^phox^, a cytosolic and a membrane component of NADPH oxidase in our present study. Consequently, the greater ROS generation in brain of mice with EPA or DHA intake could be explained. In addition, EPA or DHA decreased brain GR activity, which certainly limited the conversion of GSSG to GSH. Thus, it was reasonable to observe higher GSSG level in brain. These results revealed that EPA or DHA impaired brain GSH homeostasis. It is notified that EPA or DHA at test dose did not further affect brain expression of HO-1, cytosolic and nuclear Nrf2. Thus, the increased brain oxidative stress from EPA or DHA could be mainly attributed to these PUFAs activating NADPH oxidase and disturbing GSH homeostasis. Compared with ALA, EPA or DHA has longer chain and more unsaturated sites. Based on these structural characteristics, EPA and DHA are highly chemically unstable. That is, these two PUFAs may easily react with other factors such as oxygen, an abundant substance in brain, to initiate a series of oxidative response. It is reported that fish omega-3 fatty acids or DHA enhanced oxidative injury in brain and liver [6, 7]. Our findings also indicated that EPA and DHA exacerbated oxidative stress to brain under aging condition. The other possibility is that EPA or DHA facilitates oxidative reactions in already damaged brain, like this DG-induced aging model. These findings suggest that the supplement of antioxidant(s) might be necessary for elderly people when they take EPA and DHA supplement, or consume foods rich in EPA and/or DHA.

DG treatment increased brain AA level. AA, a lipid mediator of inflammation, could be converted to prostaglandin (PGE) and thromboxane through the action of COX [28]. It is reported that COX-2 and its metabolites, especially PGE_2_, are major pathological contributors toward the progression of brain inflammatory disorders [29]. Thus, the higher COX-2 expression and activity and PGE_2_ level in brain of DG treated mice as we observed could be ascribed to the increased AA level. Furthermore, we found that EPA or DHA intake effectively lowered brain AA content, which subsequently diminished COX-2 expression and activity, and consequently reduced PGE_2_ formation. These events suggest that EPA or DHA could decline the inflammatory pathway of AA/COX-2/PGEs in brain. On the other hand, our data regarding IL-1β, IL-6 or TNF-α levels, NF-κB p50/65 DNA binding activity and NF-κB activation indicated that EPA or DHA did not further mediate brain NF-κB related inflammatory pathway under this aging model. These findings suggested that EPA or DHA *via* altering brain fatty acid profile mildly attenuate brain inflammatory stress.

Hong *et al*. [30] reported that p53 blocks NF-kB-mediated survival signaling in ischemic brain. Chatoo *et al*. [31] indicated that p53 exhibited pro-oxidant activity in neurons, and its activation led to cell cycle arrest and apoptosis. Both p16 and p21 are cyclin-dependent kinase inhibitors, and involved in cellular senescence by initiating cell cycle arrest. The upregulation of p16 and/or p21 promotes aging in brain or other tissues [32, 33]. We found that EPA or DHA further increased DG-induced brain expression of p53, p16 and p21. These findings suggest that these two PUFAs could accelerate senescence through increasing these aging proteins. It is reported that ROS is responseible for aging signals by activating p53 and p16 pathways [34, 35]. Thus, it is highly possible that increased ROS caused by EPA or DHA subsequently stimulated the expression of these aging proteins. Since EPA or DHA intake at this dose enhanced brain oxidative stress and senescence proteins expression, the brain functions might be adversely affected.

In conclusion, the intake of EPA or DHA in DG-treated mice decreased brain LDL and declined inflammatory pathway of AA/COX-2/PGEs. However, these two PUFAs promoted brain oxidative stress and aging protein overproduction. These findings suggest that these two PUFAs have double-sided effects toward aging brain. Therefore, the safety of EPA, DHA or foods rich in these PUFAs should be carefully re-considered.
